# Real-life quantitative G6PD screening in *Plasmodium vivax* patients in the Brazilian Amazon: A cost-effectiveness analysis

**DOI:** 10.1371/journal.pntd.0010325

**Published:** 2022-03-24

**Authors:** Jose Diego Brito-Sousa, Henry M. Peixoto, Angela Devine, Alexandre V. Silva-Neto, Patricia C. S. Balieiro, Vanderson S. Sampaio, Sheila Vitor-Silva, Maxwell O. Mendes, Brenda K. A. Souza, Marcus V. G. Lacerda, Wuelton M. Monteiro

**Affiliations:** 1 Instituto de Pesquisa Clínica Carlos Borborema, Fundação de Medicina Tropical Dr Heitor Vieira Dourado, Manaus, Brazil; 2 Escola Superior de Ciências da Saúde, Universidade do Estado do Amazonas, Manaus, Brazil; 3 Faculdade de Medicina, Universidade de Brasilia, Brasilia, Brazil; 4 Global and Tropical Health Division, Menzies School of Health Research, Charles Darwin University, Darwin, NT, Australia; 5 Centre for Epidemiology and Biostatistics, Melbourne School of Population and Global Health, University of Melbourne, Melbourne, Victoria, Australia; 6 Fundação de Vigilância em Saúde Dr Rosemary Costa Pinto–FVS-RCP, Manaus, Brazil; 7 Escola de Enfermagem de Manaus, Universidade Federal do Amazonas–UFAM, Manaus, Brazil; 8 Instituto Leônidas & Maria Deane–ILMD, Fundação Oswaldo Cruz, Manaus, Brazil; Vienna, AUSTRIA

## Abstract

**Background:**

As quantitative glucose 6-phosphate dehydrogenase deficiency (G6PDd) screening tools are evaluated in operational studies, questions remain as to whether they are cost-effective. Here, a cost-effectiveness analysis (CEA) was performed to estimate the Incremental Cost-effectiveness Ratio (ICER) of the introduction of quantitative screening test to detect G6PDd among *P*. *vivax* carriers in two municipalities in the Brazilian Amazon.

**Methodology/Principal findings:**

This cost-effectiveness analysis evaluated the use of the Standard G6PD quantitative screening test in vivax malaria treatment units in two municipalities of the Brazilian Amazon. Using the perspective of the Brazilian public health system, the analysis was performed for the outcome ‘PQ-associated hospitalization avoided’, based on a decision tree model. The results indicated that the G6PDd screening strategy compared with the routine strategy was highly cost-effective, with an ICER of US$495 per additional hospitalization avoided, which represented less than 8% of one Brazilian gross domestic product per capita (US$6,822). The uncertainties evaluated in the sensitivity analysis did not significantly affect the ICER identified in the base-case.

**Conclusions/Significance:**

This cost-effectiveness analysis showed the quantitative G6PD testing was effective in avoiding PQ-associated hospitalizations. The incorporation of G6PD screening is of paramount importance towards *P*. *vivax* malaria elimination in the Amazon to promote the safe use of primaquine and tafenoquine.

## Author summary

Although relatively known for over 50 years, G6PD deficiency still greatly impairs *Plasmodium vivax* malaria treatment due to primaquine-associated hemolysis (destruction of red blood cells). Several screening platforms are now available, which can be performed without specialized equipment and personnel, before providing malaria treatment. The main question is: in an already established public health system, would they be cost-effective to avoid the deleterious effect of hemolysis? This study evaluated the real use of the Standard G6PD screening test in two small municipalities in the Brazilian Amazon. Results show that the real-life use of the test was highly cost-effective when compared to a scenario with no routine G6PD screening. Hence, the negative clinical and economic consequences associated with inadvertent prescription of PQ to patients with G6PDd were diminished.

## Introduction

Primaquine and tafenoquine are the only available drugs for the radical cure of *Plasmodium vivax malaria*. Glucose 6-phosphate dehydrogenase deficiency (G6PDd), an X-linked genetic disorder, greatly impacts malaria burden worldwide due to primaquine/tafenoquine associated-hemolysis [1]. In most vivax endemic countries, fear of triggering hemolysis has reduced or even prevented the use of 8-aminoquinolines [2]. Since no routine testing is performed in the Brazilian Amazon, where G6PDd is estimated to affect 5% of the population, primaquine (0.5mg/kg/day) is prescribed without prior knowledge of the patient’s G6PD status, which can lead to life-threatening complications, such as acute renal failure, severe anemia, and death [2–4]. In a study from the Amazon, the frequency of PQ-induced hemolysis was 85.2 cases per 100,000 PQ users [4]. Consequently, it is estimated that US$ 5,5 million a year is spent on managing cases of hemolysis in the Brazilian public health system [5]. The incorporation of G6PD screening in malaria elimination strategies is urgent [6]. Furthermore, the use of the weekly primaquine regimen, a more tolerable option for those with G6PDd due to the transient fall in hemoglobin levels within the first two doses [7], can mitigate severe clinical complications.

New point-of-care G6PD screening platforms are now available, including qualitative and quantitative options. Quantitative testing can overcome limitations of qualitative testing platforms in discriminating females with intermediate activity [8,9]. While operational challenges of using these tests have been addressed [10–12], the cost-effectiveness a screening strategy with a quantitative test at initial contact with the healthcare system has not been evaluated. Here, a cost-effectiveness analysis (CEA) was performed to estimate the incremental cost-effectiveness ratio (ICER) of the introduction of quantitative screening test to detect G6PDd among *P*. *vivax* carriers in two municipalities in the Brazilian Amazon, compared to the routine recommended by the Brazilian Ministry of Health (MoH).

## Methods

### Ethics statement

This study was approved by the Ethics Review Board at the *Fundação de Medicina Tropical Dr Heitor Vieira Dourado* in Manaus, Brazil (Study ID: 92012818.1.0000.0005).

### Study design and evaluated strategies

This cost-effectiveness analysis (CEA) was developed from the perspective of the Brazilian public health system (Sistema Único de Saúde [SUS]) for the year 2020, in two municipalities in the Brazilian Amazon that incorporated the quantitative G6PDd screening in their malaria treatment units. The analysis was performed for the outcome PQ-associated hospitalization avoided, a natural and easily interpreted outcome. To capture the main consequences of the evaluated strategies, the time horizon began at the onset of fever and ended at the end of the hospitalization period (hospitalization associated with the use of PQ).

The CEA compared two strategies, the first based on the quantitative diagnosis of G6PDd for all people diagnosed with *P*. *vivax* malaria (G6PDd screening strategy) and the second based on the routine recommended in Brazil that does not include the diagnosis of G6PDd (routine strategy). The routine strategy was based on the recommended Brazilian MoH guideline [13], which recommends the use of chloroquine for three days (10 mg/kg on day 1 and 7.5 mg/kg on days 2 and 3) and PQ for seven days (0.5 mg/kg/day), without the obligation of G6PDd screening before the indication of treatment.

The G6PDd screening strategy utilized the Standard G6PD quantitative test (SD Biosensor, South Korea) to diagnose G6PDd in patients infected with *P*. *vivax* before the use of antimalarial treatment. Patients with activity < 30% of normal (<4 IU/gHb) were diagnosed as G6PD deficient. They were treated with chloroquine for three days (10 mg/kg on day 1 and 7.5 mg/kg on days 2 and 3) and supervised weekly PQ (0.75mg/kg/week for eight weeks). Patients with enzyme activity ≥30% of normal (≥ 4 IU/gHb) underwent treatment with chloroquine for three days (10 mg/kg on day 1 and 7.5 mg/kg on days 2 and 3) and primaquine (PQ) for seven days (0.5 mg/kg/day).

### Target population

The analysis was carried out in the municipalities Mâncio Lima (State of Acre) and Rio Preto da Eva (State of Amazonas), both located in the Brazilian Amazon. The municipality of Mâncio Lima is located 670 km from the capital Rio Branco and has an estimated population of 19,311 inhabitants. The municipality of Rio Preto da Eva, with an estimated population of 33,347 inhabitants, is located just 78 km from the capital Manaus. In both municipalities, malaria diagnosis is made by thick blood smear (TBS). While both qualitative and quantitative G6PDd screening were implemented within the scope of the Safeprim study [10,14], only the quantitative screening strategy was considered for the cost-effectiveness analysis. The quantitative screening strategy started for both municipalities in January 2020.

### Analytical decision tree model

**Fig 1:**
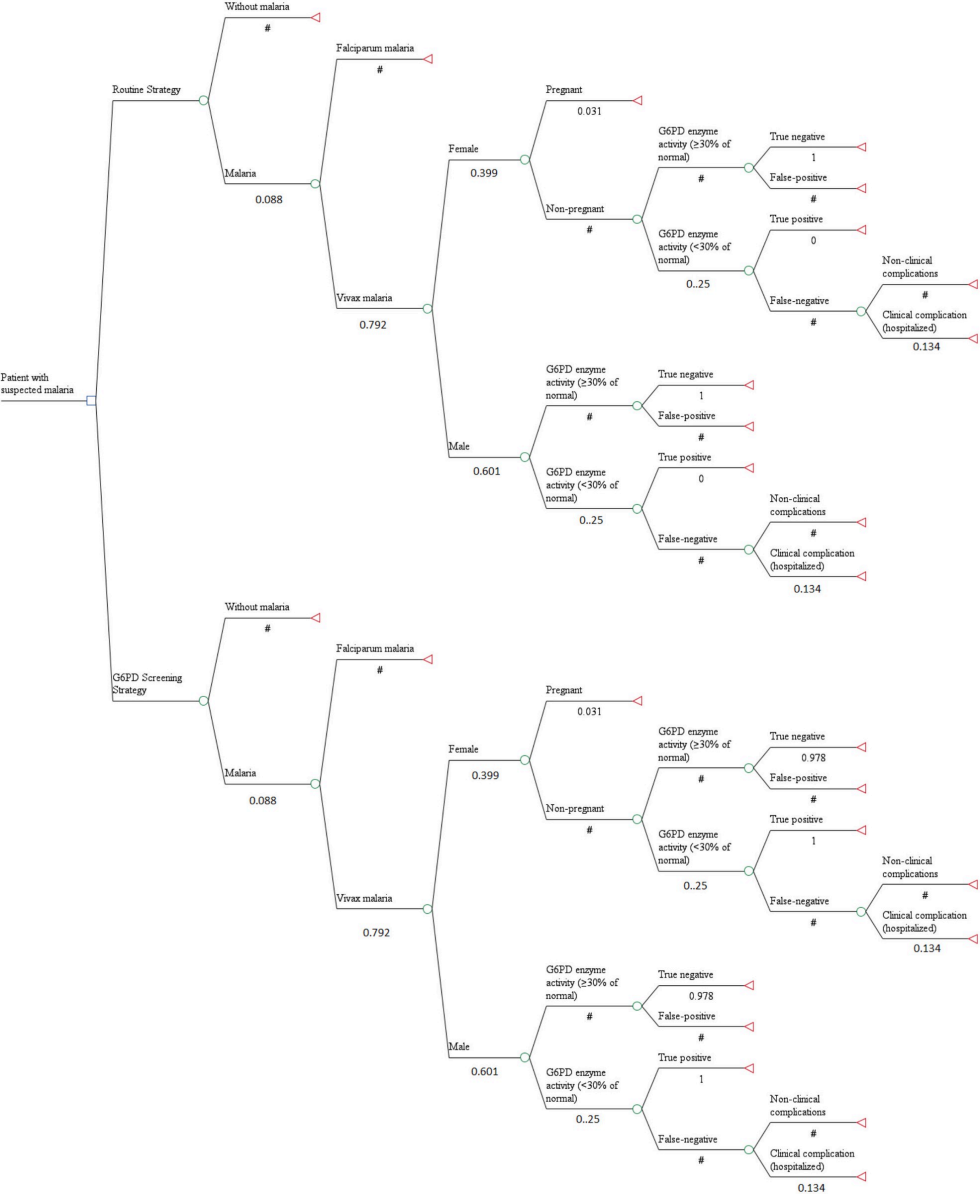
Analytical decision tree model used for cost-effectiveness analysis.

A decision tree model was built to compare the strategies under evaluation (Fig 1). After the initial decision node with the two strategies, probability nodes were created based on the suspicion of malaria. The patient with suspected malaria was subjected to the probability of having or not malaria, followed by the probability of whether malaria was caused by *P*. *vivax* and whether the patient was male or female. For the female patient infected with *P*. *vivax*, the probability of pregnancy was also considered. After the diagnosis of vivax malaria, both sexes of patients were given probabilities of having G6PD enzyme activity lower or higher than 30% and then to the probabilities of being adequately classified by the quantitative test (sensitivity and specificity). Finally, the probability of hospitalization due to the use of PQ was considered. For the terminal node, a value of 1 was assigned when hospitalization associated with the use of PQ was avoided and 0 when the hospitalization associated with the use of PQ was not avoided.

### Epidemiological parameters

The epidemiological parameters relating to cases of malaria and G6PDd were obtained from the Malaria Epidemiological Surveillance Information System (SIVEP/Malaria) of the municipalities [15], and the results obtained in the Safeprim operational study [14] which implemented the use of the Standard G6PD quantitative test for all patients with vivax malaria in the municipalities considered in this study. According to the Safeprim study [14], the test identified 66 patients (40 males and 26 females) with enzyme activity below 30% of normal out of 1,643 *P*. *vivax* malaria patients, 41 in Mâncio Lima (25 males and 16 females, January to December 2020) and 25 in Rio Preto da Eva (15 males and 10 females, January to August 2020) among 1,231 and 412 patients, respectively. Data from the scientific literature were used to complement the information needed for the model (Table 1).

**Table 1:** Epidemiological parameters considered in the analytical model for the Brazilian Amazon.

Parameter	Base-case	Variation	Distribution	Source
Proportion of febrile patients given a malaria test who test positive for malaria	0.088^a^	(0.078–0.170)^b^	Beta (α: 2,503 and β: 25,903)	[15]
Proportion of malaria patients who have vivax malaria	0.792^a^	(0.738–0.994)^b^	Beta (α: 1,982 and β:521)	[15]
Proportion of male patients with vivax malaria among all male patients with vivax malaria	0.601^a^	(0.601–0.602)^b^	Beta (α:1,191 and β: 791)	[15]
Proportion of female vivax malaria patients who are pregnant	0.031^a^	(0.028–0.036)^b^	Beta (α:785 and β: 24,508)	[15,16]
Proportion of female patients with vivax malaria who have G6PD enzyme activity less than 30% of normal	0.025^a^	(0.020–0.039)^b^	Beta (α: 16 and β: 637)	[14]
Proportion of male patients with vivax malaria who have G6PD enzyme activity less than 30% of normal	0.025^a^	(0.021–0.037)^b^	Beta (α: 25 and β: 965)	[14]
Sensitivity (SD Biosensor Standard G6PD test)	1.00	(0.938–1.00)^c^	Beta (α: 58 and β: 1)	[8]
Specificity (SD Biosensor Standard G6PD test)	0.978	(0.970–0.985)^c^	Beta (α: 1,599 and β: 36)	[8]
Proportion of patients with enzyme activity less than 30% of normal hospitalized after treatment with PQ	0.134^d^	(0. 089–0.162)^d^	Beta (α:94 and β: 606)	[4,14]

a: parameter calculated based on data from both municipalities

b: calculated variation considering the data from the municipalities separately, represents the variation between the two assessed municipalities

c- variation based on the 95% confidence interval of the measure

d: Proportion estimated based on data from the literature and from the Safeprim study (S1 Table).

### Cost parameters

Direct medical costs arising from the diagnosis of malaria, diagnosis of G6PDd, vivax malaria treatment and costs of care provided to serious adverse events associated with the use of PQ in patients with G6PDd were included in the analysis (Table 1). Information from the Safeprim study [14], the Brazilian Hospital Information System (SIH/SUS) and the literature were used (Table 2).

**Table 2:** Cost components and unit costs considered in the analytical model for the Brazilian Amazon (2020 US$).

Items	Base-case	Variation	Distribution	Source
***P*. *vivax* diagnosis**				
Thick blood smear	0.808	0.166–1.238^e^	Triangular (min: 0.166; max: 1.238)	[19,20]
Microscope	0.461	0.370–0.553^e^	Triangular (min: 0.370; max: 0.553)	[19,20]
Microscope maintenance	0.034	0.029–0.043^e^	Triangular (min: 0.029; max: 0.043)	[19,20]
Health workers	2.154	1.722–2.584^e^	Triangular (min: 1.722; max: 2.584)	[19,20]
Yearly training (microscopist)	3.506	2.805–4.208^e^	Triangular (min: 2.805; max: 4.208)	[19,20]
**Treatment scheme**				
CQ 3 days + PQ 7 days^a^	0.173	0.138–0.208^f^	Triangular (min: 0.138; max: 0.208)	[21]
CQ 3 days + weekly supervised PQ^b^	0.256	0.205–0.307 ^f^	Triangular (min: 0.205; max: 0.307)	[21]
CQ 3 days + weekly CQ^c^	0.403	0.322–0.484^f^	Triangular (min: 0.322; max: 0.484)	[21]
**G6PDd diagnosis** ^**d**^				
- Training	0.358	0.286–0.430^f^	Triangular (min: 0.286; max: 0.430)	[14]
- Health worker	1.077	0.862–1.292^e^	Triangular (min: 0.862; max: 1.292)	[19,20]
- Test kit cost (SD Biosensor Standard G6PD test)–single test	8.728	6.982–10.474^f^	Triangular (min: 6.982; max: 10.474)	[14]
- Supplies (SD Biosensor Standard G6PD test)—single test	0.224	0.184–0.330^g^	Triangular (min: 0.184; max: 0.330)	[14,22]
Analyzer (SD Biosensor Standard G6PD test)–single test	2.436	1.593–4.733^g^	Triangular (min: 1.593; max: 4.733)	[14]
**Medical assistance to the G6PDd patient treated with PQ**				
Pre-admission tests	6.615	5.973–7.044^e^	Triangular (min: 5.973; max: 7.044)	[19,20]
Medical appointments	2.865	-		[23]
Hospitalization (AIH)	123.022	98.417–147.626^e^	Gamma (α: 1.2963; ʎ: 0.0105)	[19,20]

a: treatment indicated for patients with G6PD enzyme activity greater than or equal to 30% of normal

b: treatment (0.75mg/kg/week PQ per 8 weeks, supervised) indicated for patients with G6PD enzyme activity less than 30% of normal

c: treatment indicated for pregnant patients

d: G6PDd diagnosis costs detailed in S1 Box

e: variation identified in economic studies previously published by our research group

f: variation of +/-20% from base-case

g: variation calculated considering the data from the municipalities separately (number of cases, number of health units and equipment used), represents the variation between the two municipalities evaluated.

Costs obtained in Brazilian currency (reais, R$) were converted into US dollars (US$) using the average official exchange rate for the year 2020 (R$5.16 per dollar) [17]. The values of costs identified in years other than the year evaluated were adjusted based on the official inflation rate estimated by the Extended National Consumer Price Index (IPCA) in Brazil [18]. Since the time horizon was less than one year, discount rates were not used for the costs or outcomes.

### Cost-effectiveness and sensitivity analysis

The model incorporated the epidemiological parameters and costs to calculate the costs and PQ-associated hospitalization avoided of both strategies under evaluation as well as the calculation of the incremental cost-effectiveness ratio (ICER) of implementing G6PD screening as compared to usual practice. TreeAge Pro Healthcare 2021 Software was used to build the decision model, calculate the base-case ICER and for all sensitivity analyses.

To assess the impact of uncertainty related to epidemiological parameters and costs, univariate and multivariate sensitivity analyzes were performed. The results of the univariate analysis were presented using a tornado diagram, which deterministically expressed the individual impact of the variables on the ICER. For the multivariate evaluation, a probabilistic sensitivity analysis (PSA) was conducted using second-order Monte Carlo simulation, which evaluated the impact of uncertainty for all parameters on the ICER using 10,000 model interactions. For the PSA input parameters, the beta distribution was used for the probability parameters and gamma distribution, when it was possible to estimate alpha and lambda, or triangular distribution for the cost parameters (Tables 1 and 2). PSA was presented using the acceptability curve based on willingness-to-pay of US$6,822 (one gross domestic product (GDP) *per capita* for Brazil) for additionally PQ-associated hospitalization avoided.

The present analysis used the same assumptions described by Peixoto et al [5] for the diagnosis of malaria and by Peixoto et al [19] for the assistance provided to G6PDd carriers hospitalized after a serious adverse event associated with PQ. Based on unpublished information from the Safeprim study, we assumed that patients treated with weekly supervised PQ were not hospitalized due to serious adverse events associated with PQ.

## Results

The cost-effectiveness analysis presented in Table 3 indicates that the G6PDd screening strategy, based on the Standard G6PD quantitative test, when compared to the Routine strategy, presented an ICER of US$495.32 per additional PQ-associated hospitalization avoided.

**Table 3:** Cost-effectiveness results (per person) for the base-case analysis in the Brazilian Amazon in 2020 United States Dollars (US$).

Strategy	Effectiveness^a^	Incremental effectiveness	Cost (US$)	Incremental cost (US$)	ICER^b^ (US$ /incremental outcome)
Routine strategy	0	-	7.0058	-	-
G6PDd screening strategy	0.00172	0.00172	7.8582	0.8524	495.32

a. Effectiveness for the outcome PQ-associated hospitalization avoided

b. ICER, incremental cost-effectiveness ratio.

**Fig 2:**
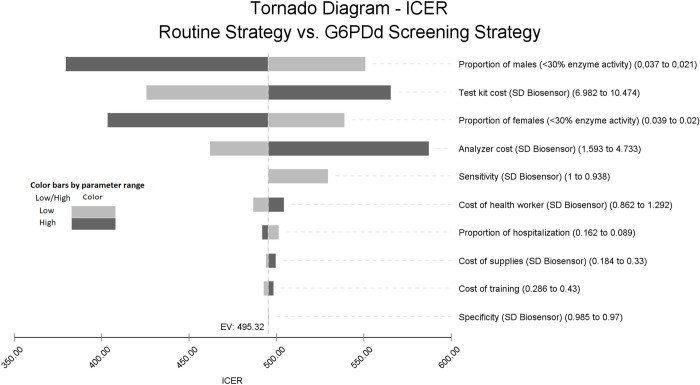
Incremental Cost-effectiveness Ratio (ICER) Tornado diagram for univariate sensitivity analysis of routine strategy versus G6PDd screening strategy, based on the individual impact of each of the variables on the ICER. The vertical line denotes the base-case expected value (EV).

The tornado diagram presents the univariate sensitivity analysis based on the epidemiological and cost parameters that most impacted the ICER (Fig 2). The upper limit of the proportion of male patients with G6PD enzyme activity less than 30% estimated the lowest ICER (US$379.35/ additional PQ-associated hospitalization averted), and the upper limit of the weighted cost for an analyzer test estimated the highest ICER (US$587.20/ additional PQ-associated hospitalization averted). These results remained reasonably similar to the ICER identified for the base-case (US$495.32).

**Fig 3:**
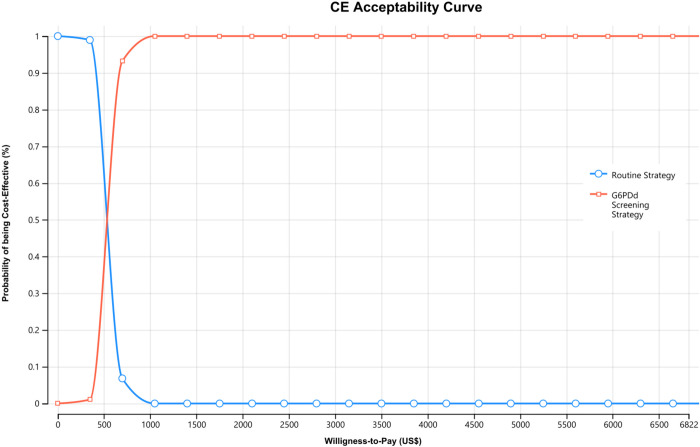
Cost-effectiveness acceptability curve of routine strategy versus G6PDd screening strategy. Brazilian Amazon, 2020.

The PSA resulted in an average ICER of US$539.62 (95% CI 537.67–541.57) per additional PQ-associated hospitalization avoided, a result similar to the base case. The cost-effectiveness acceptability curve shown in Fig 3 expresses the PSA results. At values of US$1,050 and higher, the G6PDd screening strategy had a 100% probability of being cost-effective. US$1,050 corresponds to 15.4% of one GDP per capita in Brazil (US$6,822), which is commonly used as the willingness-to-pay threshold for a quality-adjusted life-year gained, disability-adjusted life-year averted and for outcomes related to hospitalizations in the context of neglected diseases in Brazil.

## Discussion

This CEA is part of a larger research project, the Safeprim study, which studied the real-life implementation of a quantitative screening test to detect G6PDd among individuals infected with *P*. *vivax* in two municipalities located in the Brazilian Amazon. The results indicated that the G6PDd screening strategy compared with the routine strategy without screening was highly cost-effective, with an ICER of US$495.32 per additional PQ-associated hospitalization avoided. Furthermore, the G6PDd screening strategy avoided not only the costs associated with each hospitalization avoided, but also all the negative clinical and economic consequences associated with inadvertent prescription of PQ to patients with G6PDd, providing safe and ethical care that was previously unavailable to patients with this enzymopathy.

The ICER of US$495.32 per additional PQ-associated hospitalization avoided is less than 8% of one Brazilian GDP per capita (US$6,822). Although there is no explicit value to the cost-effectiveness threshold for a very efficient intervention in Brazil [24], CEAs conducted in the Brazilian context of neglected diseases have adopted interventions with ICERs up to one Brazilian GDP per capita for each additional unit of the health effect, including: hospitalization averted [19], days of hospitalisation avoided [25], case averted [26], and cure [27]. These outcomes are natural units that are intuitively understood in the health care field, facilitating decision making [28]. Accordingly, the choice of a cost-effectiveness threshold for a hospitalization avoided were based on suggestions such as that proposed by the WHO, which indicates that a strategy capable of avoiding a disability-adjusted life-year at less than one GDP per capita should be very cost-effective [29], although the WHO later clarifies that this is not a recommendation [30], and by Pichon-Rivière, who recommended a threshold of 0.62–1.05 GDP per capita per quality-adjusted life-year gained in Brazil [31].

Both municipalities evaluated are considered endemic for malaria, with a predominance of vivax malaria. However, the municipalities showed differences in risk of malaria caused by *P*. *vivax*. For example, the incidence rate in Mâncio Lima was 75.71 cases per 1,000 inhabitants as compared to 15.60 cases in Rio Preto da Eva. In the cost-effectiveness analysis for the base case, we aimed to present parameters that would reflect the average across both municipalities, while the sensitivity analysis reflects the epidemiological differences identified in the municipalities.

In this scope, we performed univariate sensitivity analyzes and did not identify important impacts on the ICER (Fig 2). Thus, the G6PDd screening strategy remained efficient even when considering the different epidemiological scenarios and other uncertainties (Tables 1 and 2). These results were corroborated by PSA, which indicated a 100% probability of the G6PDd screening strategy being cost-effective with only 15.4% willingness-to-pay.

Previous CEAs have already demonstrated that strategies based on the use of the qualitative rapid diagnostic test (CareStart G6PD) were cost-effective [19,32], including in the Brazilian Amazon. Other studies have indicated that the strategy based on CareStart G6PD were cost-effective in avoiding hospitalization and showed a negative incremental budget impact [33]. Our study demonstrates that quantitative testing with SD biosensor remains cost-effective while including technological advantages, such as the important gains in diagnostic accuracy.

Previous research has demonstrated that health care professionals can reliably perform G6PD tests even with no previous knowledge on G6PDd deficiency [10,12]. One study assessed the use of the CareStart qualitative test in malaria treatment units, finding that most professionals were able to correctly use the test after a single training section [10]. However, limitations on test specificity led to loss of opportunities for proper radical cure treatment since many patients received weekly rather than daily PQ due to a false deficient result. Little is currently known about the effectiveness of weekly PQ used in real-life settings. Due to their good performance and ability to identify individuals with intermediate activity [8,34], quantitative testing platforms, such as the Standard biosensor, have now advanced to operational studies in pragmatic conditions.

Tafenoquine, an 8-aminoquinoline with longer half-life than primaquine (mean 12 days) [35], was recently approved by Brazilian regulatory agencies. Its use requires greater caution compared to primaquine, and requires quantitative G6PD screening since only individuals with > 70% activity are eligible to receive tafenoquine [36]. Even those who will not be prescribed tafenoquine will benefit from radical cure in Brazil, as patients with G6PD activity of 30–70% will receive 7-day primaquine and those with <30% activity will receive weekly primaquine. Sex-guided treatment strategies may potentially ensure widespread use of tafenoquine for radical cure in low resource settings [37]. Future research should examine the ICER of tafenoquine.

This study has a number of limitations related to the parameter values. Operational issues did not enable the identification the proportion of patients with enzyme activity less than 30% of normal hospitalized after treatment with PQ based on exclusive data from the evaluated municipalities. In addition, some cost items related to diagnosis and treatment of malaria were not available. To mitigate these limitations, the study estimated the referred proportion and costs based on previous studies carried out in the Brazilian Amazon. The univariate sensitivity analysis, however, showed that those uncertainties did not significantly affect the ICER.

In conclusion, quantitative G6PD testing was effective in avoiding PQ-associated hospitalizations whilst remaining cost-effective. Policy *makers should* consider the incorporation of G6PD screening towards *P*. *vivax* malaria elimination in the Amazon to promote safe and ethical use of primaquine and tafenoquine.

## Supporting information

S1 Table Calculation of the proportion of patients with enzyme activity less than 30% of normal hospitalized after treatment with primaquine for seven days. (DOCX)

S1 Box Details of the costing of the items considered for the G6PDd diagnosis. (DOCX)
