# Supplementary material for: Real-life quantitative G6PD screening in Plasmodium vivax patients in the Brazilian Amazon: A cost-effectiveness analysis
Source: PLoS Negl Trop Dis. 2022 Mar 24;16(3):e0010325. doi: 10.1371/journal.pntd.0010325 (PMC8982881; doi:10.1371/journal.pntd.0010325)
Supplement: S1 Table — (DOCX) [file pntd.0010325.s001.docx]

**S1 table.** Calculation of the proportion of patients with enzyme activity less than 30% of normal hospitalized after treatment with primaquine for seven days.

| **Variable** | **data/estimates** | **Source** |
| --- | --- | --- |
| Total *P. vivax* malaria patients | **A:** 28095 | [1] |
| Proportion of patients with enzyme activity less than 30%^a^ , base case (variation)^b^ | **B:** 0.02491  (0.02065-0.037621) | [2] |
| Estimated number of patients with enzyme activity less than 30% | **C=**A*B= 699.72 (580.16 – 1056.97) | - |
| Hospitalizations in G6PDd patients | **D:** 94 | [1] |
| Proportion of hospitalization (base case) | **E:** D/C=**0.134** | - |
| Proportion of hospitalization (variation)^c^ | **0.089-0.162** | - |

^a^ Proportion estimated based on data from the Safeprim study; b. The proportion for the base case was estimated based on data from both municipalities. For the variance, the data from the municipalities were considered separately. C. Base case was estimated based on the proportion of patients with enzyme activity less than 30% estimated for both municipalities. To estimate the variance, the data from the municipalities were considered separately.

References

1. Brito-Sousa JD, Santos TC, Avalos S, Fontecha G, Melo GC, Val F, et al. Clinical Spectrum of Primaquine-induced Hemolysis in Glucose-6-Phosphate Dehydrogenase Deficiency: A 9-Year Hospitalization-based Study From the Brazilian Amazon. Clin Infect Dis. 2019;69: 1440–1442. doi:10.1093/cid/ciz122

2. Fundação de Medicina Tropical Dr. Heitor Vieira Dourado. Avaliação implementação de teste para diagnóstico de deficiência de enzima G6PD - SAFEPRIM [Internet]. 2020. p. 0. Available: https://www.vivaxmalaria.org/implementation-of-rapid-tests-for-diagnosis-of-glucose-6-phosphate-dehydrogenase-deficiency-in
