# Supplementary material for: Real-life quantitative G6PD screening in Plasmodium vivax patients in the Brazilian Amazon: A cost-effectiveness analysis
Source: PLoS Negl Trop Dis. 2022 Mar 24;16(3):e0010325. doi: 10.1371/journal.pntd.0010325 (PMC8982881; doi:10.1371/journal.pntd.0010325)
Supplement: S1 Box — (DOCX) [file pntd.0010325.s002.docx]

**S1 box 1. Details of the costing of the items considered for the G6PDd diagnosis.**

| **Analyzer cost (SD Biosensor Standard G6PD test) – single test (US$)** | | | | | | | | | | | | **Source** [1] |
| --- | --- | --- | --- | --- | --- | --- | --- | --- | --- | --- | --- | --- |
| Value of an Analyzer | (A) Annualized value of an analyzer | | (B) Average number of tests performed per analyzer | | | | (C) Cost per test  C = A/B  Base-case (variation) | | | | | |
| 717.06^a^ | 165.64^b^ | | 68^c^ | | | | - 1. (0.184- 0.330)^d^ | | | | | |
| **Test kit cost (SD Biosensor Standard G6PD test) – single test (US$)** | | | | | | | | | | | **Ref.** [1] | |
| (A) Test kit | (B) Number of tests per kit | | | (C) Cost per test  C=A/B  Base-case (variation) | | | | | | | | |
| 218.201^a^ | 25 | | | 8,728 (6.982-10.474)^f^ | | | | | | | | |
| **Supplies costs (SD Biosensor Standard G6PD test) - single test (US$)** | | | | | | | | | | **Source** [1,2] | | |
| Supplies per test | | | | | | Cost per test | | | | | | |
| Additional supplies (e.g. lancet, cotton, gloves, lcohol) | | | | | | 0.110 | | | | | | |
| Control Kit | | | | | | 0.113^g^ | | | | | | |
| Total: base-case (variation) - Single test | | | | | | 0.224 (0.184-0.330)^d^ | | | | | | |
| **Training cost (US$)** | | | | | | | | | **Source** [1] | | | |
| Itens | | Cost per training | | | (F) Number of tests performed | | | (G) Cost per test  G=E/F  Base-case (variation) | | | | |
| (A) Instructor | | 310,33^h^ | | |  | | |  | | | | |
| (B) Educational material (e.g pen, paper, folder, poster) | | 5,605 | | |  |  |  |  |  |  |  |  |
| (C) Snacks for the participants | | 38,791 | | |  |  |  |  |  |  |  |  |
| (D) Total costs per training (A+B+C) | | 354,727 | | |  |  |  |  |  |  |  |  |
| (E) Total costs for two training E=(D*2) | | 709.453 | | | 1982 | | | 0.358 (0.286-0.430)^f^ | | | | |

a. Value provided to Safeprim study by the manufacturer; b. Cost per year was calculated by dividing the value of the equipment by an annualization factor (4.329), which considered an estimated usage time of 5 years (based on the opinion of healthcare workers who use similar equipment in SUS) and a discount rate of 5%. C. Ratio between the estimated number of tests performed and the number of analyzers used by the municipalities (1983/29) d. Base-case calculation based on data from both municipalities and variation based on data from the municipalities separately; f. Variation of +/- 20% from base-case; g. Kit controls cost for 20 tests (USS$9.50) divided by 20, multiplied by the average number of controls per analyzer in one year (16.22), divided by the average number of tests per equipment in one year (68); h. Based on a training of 4 hours by 4 instructors. The following costs were considered: one day of work per instructor. food, travel, and accommodation.

References

1. Fundação de Medicina Tropical Dr. Heitor Vieira Dourado. Avaliação implementação de teste para diagnóstico de deficiência de enzima G6PD - SAFEPRIM [Internet]. 2020. p. 0. Available: https://www.vivaxmalaria.org/implementation-of-rapid-tests-for-diagnosis-of-glucose-6-phosphate-dehydrogenase-deficiency-in

2. Ministério da Saúde do Brasil. Banco de Preços em Saúde [Internet]. 2020.
